# Coordination of Gene Expression and Growth-Rate in Natural Populations of Budding Yeast

**DOI:** 10.1371/journal.pone.0088801

**Published:** 2014-02-12

**Authors:** Zvi Tamari, Dalia Rosin, Yoav Voichek, Naama Barkai

**Affiliations:** Department of Molecular Genetics, Weizmann Institute of Science, Rehovot, Israel; University of Cambridge, United Kingdom

## Abstract

Cells adapt to environmental changes through genetic mutations that stabilize novel phenotypes. Often, this adaptation involves regulatory changes which modulate gene expression. In the budding yeast, ribosomal-related gene expression correlates with cell growth rate across different environments. To examine whether the same relationship between gene expression and growth rate is observed also across natural populations, we measured gene expression, growth rate and ethanol production of twenty-four wild type yeast strains originating from diverse habitats, grown on the pentose sugar xylulose. We found that expression of ribosome-related genes did not correlate with growth rate. Rather, growth rate was correlated with the expression of amino acid biosynthesis genes. Searching other databases, we observed a similar correlation between growth rate and amino-acid biosyntehsis genes in a library of gene deletions. We discuss the implications of our results for understanding how cells coordinate their translation capacity with available nutrient resources.

## Introduction

Phenotypic diversity results from genetic changes that modify gene function or gene expression. In some cases, a phenotype can be clearly traced back to the genotype, for example when a specific metabolic enzyme is lost. More often, however, phenotypic differences are harder to explain based on the available genotypes, particularly when comparing related strains or species, in which case, phenotypic differences likely arise from changes in gene regulation [1]. Genome-wide expression profiling revealed significant differences in the transcription program of closely related strains or species, but the phenotypic consequences of the majority of the expression differences remains poorly understood.

Budding yeast provide a promising model for studying the relationship between gene expression and cellular phenotype. Mapping of the yeast expression program under a wide spectrum of conditions revealed a tight link to major phenotypic aspects, including growth rate and metabolic fluxes. Cell growth rate, for example, can be predicted from the expression of ribosome-associated genes [2], [3], [4], [5], including genes coding for ribosomal proteins (RP) and ribosomal RNA biogenesis (Ribi). Expression of genes associated with the environmental stress response (ESR) also correlates with cell growth rate [6], [7], [8], [9], [10], [11]. Similarly, metabolic genes involved in respiratory functions are repressed when cells are presented with a fermentable carbon source, but their expression increases when cells respire. However, whether phenotypic diversity among related budding yeast strains and species is similarly reflected in their gene expression program is not known.

Recent work revealed certain aspects of gene expression modulation that are conserved across different environments and across related strains. First, genes whose expression is highly responsive to changes in the environment diverge more rapidly between strains or species [12], [13]. This set of rapidly-diverging genes is characterized by a so-called Occupied Proximal Nucleosome promoter structure that lacks a defined nucleosome-free region and displays a TATA binding site [14], [15]. In addition, functionally related genes tend to change in a coordinated manner, both when compared across environments and when compared between related strains or species [12], [16], most likely reflecting the abundance of trans-mutations affecting environmentally-sensitive regulators [15].

To examine whether expression-phenotype relationships that hold across different environmental conditions are maintained also when comparing related strains or species, we measured gene expression, growth rate and ethanol production of twenty four natural isolates of the budding yeast. We first provided cells with glucose, their optimal carbon source. Under these conditions, diversity in growth rate and ethanol production was rather limited, thereby limiting the ability to define any correlation with gene expression.

To increase phenotypic diversity, we provided cells with the naturally-rare pentose xylulose. We observed no inter-strain correlation between growth rate and ribosome-related gene expression. Examining for other gene groups whose expression correlated with growth rate, we identified the amino-acid biosynthesis gene group, the expression of which was inversely correlated with growth rate on xylulose. This correlation was also observed when examining a large dataset of deletion mutants and drug perturbations, but not detected when growth rate was varied through growth in multiple environments.

Together, our results show that major phenotypic differences between related strains are not reflected in their gene expression program, suggesting that regulatory mechanisms triggered when cells are shifted between environments are distinct from regulatory changes selected during long-term evolution.

## Results

### Inter-strain diversity in growth increases when cells are grown on xylulose

We previously mapped the expression differences between representative strains of *S. cerevisiae* and *S. paradoxus* [12], [15]. In this study, we extended this analysis by collecting twenty-four fully sequenced budding yeast strains collected from diverse habitats and geographical origins (**Table S1**). To increase diversity, we considered twelve strains classified as *S. cerevisiae*, and twelve classified as *S. paradoxus*. The two species diverged ca. 5 MYrs ago, but are phenotypically similar and retained practically the same set of genes, with 85% and 70% average identity in coding and non-coding regions, respectively. The inter-strain sequence polymorphisms range from about 1 to 10 mutations per 1000 base-pairs.

**Figure 1:**
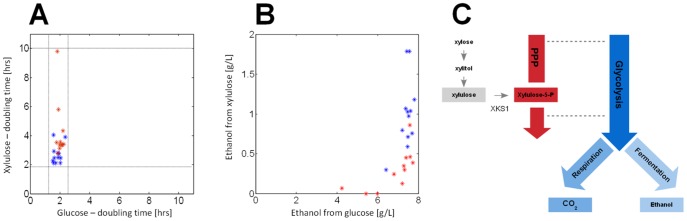
Phenotypic diversity during growth on glucose or xylulose. (A,B) Doubling times (A) and ethanol production levels (B) of all wt *S. cerevisiae* (blue) and *S. paradoxus* (red) yeast strains in our collection during growth on glucose vs. growth on xylulose. Dashed lines in (A) delimit the boundaries of doubling times for each carbon source. (C) Schematic representation of xylulose metabolism. Intracellular xylulose is phosphorylated to xylulose-5-phsophate, an intermediate of the PPP. The sugar phosphate is metabolized via the PPP and subsequently enters glycolysis through two common metabolic intermediates shared by these pathways – Fructose-6-phosphate and Glyceraldehyde-3-phosphate.

We began by examining growth on rich media containing glucose, the optimal carbon source for budding yeast. All strains grew rapidly, with little inter-strain variation in growth rate (std/mean = 0.13), and produced ethanol at a level close to the theoretical maximum (**Fig. 1A,B** and **Table S1**). The lack of diversity between strains during growth on glucose limits the ability to reliably define any relationship between growth rate and gene expression. We therefore sought for conditions in which inter-strain phenotypic variation is more pronounced.

Budding yeast cannot utilize pentoses such as xylose or arabinose, due to insufficient activities of the enzymes converting them into xylulose-5-Phosphate, an intermediate of the pentose phosphate pathway (PPP). However, yeast are able to grow if provided with xylulose as the sole carbon source, as the free sugar is phosphorylated by xylulokinase, encoded by the gene XKS1, to give the PPP intermediate (**Fig. 1C**). As xylulose is not freely available in nature, we expected it to expose wider phenotypic diversity between the strains.

Growing yeast on rich media containing pure xylulose is unpractical, due to the high cost of the pure sugar. We therefore implemented a method of xylulose production in the lab [17], yielding a mixture of xylulose and xylose in a ∼30∶70 ratio (Materials and Methods). As expected, none of the strains utilized xylose, but all efficiently metabolized the xylulose fraction, as verified by HPLC analysis of the growth media.

Growth rates, cell size and the amount of ethanol produced by each strain during growth on xylulose were characterized. As expected, growth rates on xylulose were slower than on glucose and varied considerably between strains (std/mean = 0.29). Similarly, cell size was reduced on xylulose compared to glucose (**Fig. S1**). A significant increase in diversity was also seen in the level of ethanol produced (**Fig. 1**).

### Growth on xylulose induces respiratory genes, independently of the fermentation capacity

Studies on budding yeast revealed a strong correlation between the mode of metabolism and metabolic gene expression when compared across different environments [18]. For example, during growth on glucose, a large set of respiration-specific genes is down-regulated while fermentation-specific genes are strongly expressed, consistent with the fact that glucose is fermented at practically the theoretical yield. Respiratory genes are also repressed during growth on other fermentable carbon sources, such as galactose or fructose, albeit to a lesser extent, but become strongly induced during growth on non-fermentable carbon sources, such as ethanol or glycerol, which are metabolized by respiration [19].

**Figure 2:**
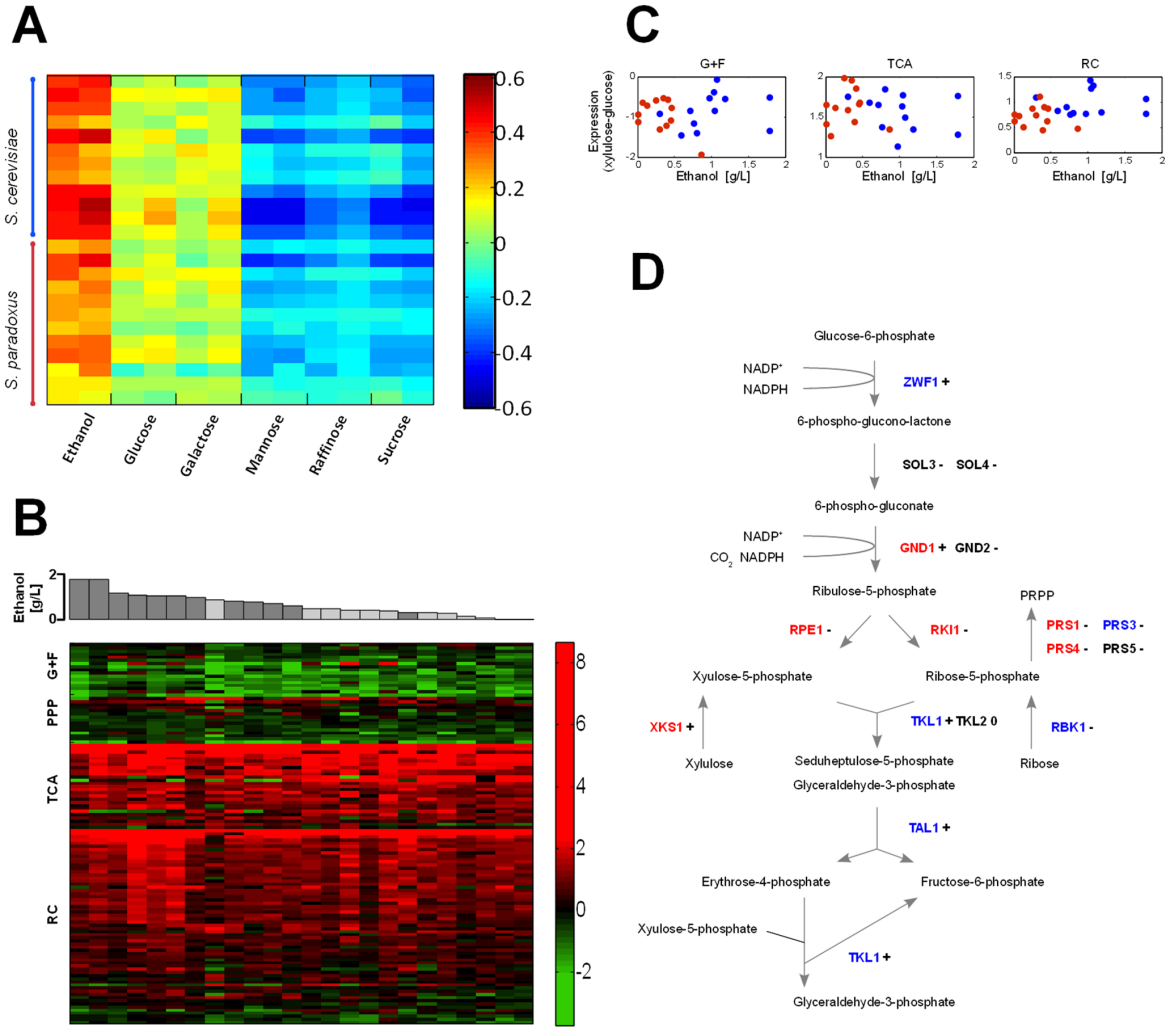
Mode of metabolism during growth on xylulose. (A) Mean expression of all genes of each of the 24 strains from our collection grown on xylulose, compared to existing expression data for a wild type yeast strain grown on various carbon sources. Colors represent the Pearson correlation coefficient for each of these comparisons. (B) Difference (log ratio) between expression on xylulose and on glucose of genes participating in glycolysis and fermentation (G+F), the PPP, the TCA cycle and the respiratory chain (RC). Genes for which data from less than 14 strains existed were omitted. The complete list of genes can be found in **Table S2**. Each column represents data from an individual strain. Ethanol production level on xylulose of the respective strain is shown above, for each *S. cerevisiae* (dark gray) and *S. paradoxus* (light gray) strain. The columns are sorted according to the level of ethanol production. Same format matrices but for absolute expression levels on glucose and xylulose are shown in **Fig. S2A** and **Fig. S3A**, respectively. (C) Mean difference (log ratio) between expression on xylulose and on glucose over all genes participating in glycolysis and fermentation (G+F), the TCA cycle and the respiratory chain (RC) for each of the 12 *S. cerevisiae* (blue) and 12 *S. paradoxus* (red) strains, vs. ethanol production levels. Same plots but for absolute expression levels on glucose and xylulose are shown in **Fig. S2B** and **Fig. S3B**, respectively. (D) Detailed description of the PPP including metabolic intermediates and genes encoding for the enzymes participating in the pathway. The color of the gene name indicates whether its expression (absolute level) on xylulose across all 24 strains is positively correlated (blue), negatively correlated (red) or not correlated (black) with the level of ethanol production (using a correlation threshold of c = ±0.3). Induction/reduction in expression of each gene on xylulose, compared to glucose, averaged over all strains, is indicated by **+** (induction), **-** (reduction) and **0** (equal expression) signs (using a log-difference threshold of ±0.2).

We mapped the transcription profiles of all twenty-four strains growing on glucose or xylulose using custom-designed Agilent microarrays based on the *S. cerevisiae* and *S. paraodoxus* reference genomes and compared the overall expression profile of each strain growing on xylulose to existing expression data of a laboratory yeast strain grown on various carbon sources [7] (**Fig. 2a**). Interestingly, the expression profile of all strains was significantly more similar to that of cells growing on the non-fermentable carbon source ethanol than to the fermentable sugar glucose, or even to the expression profile of cells growing on the fermentable but non-repressing carbon sources galactose or mannose. Focusing on metabolic genes, we observe a marked induction of respiratory genes, including genes of the TCA cycle, oxidative phosphorylation and others (**Fig. 2B**). In contrast, genes involved in glycolysis, as well as fermentation-specific genes, were repressed.

Some of the strains used in our study produced no detectable amount of ethanol when grown on xylulose, while others produced considerable amounts, up to ∼25% of that produced on the same-concentration glucose (**Fig. 1B** and **Table S1**). We asked whether this difference in xylulose fermentation capacity is reflected in the expression of metabolic genes, reasoning that better fermentation may be due to stronger repression of respiratory genes, or an induction of fermentation-specific genes. This, however, was not the case. Neither set of genes was correlated with the amount of ethanol produced (**Fig. 2B,C**). The gene expression profile of cells growing on xylulose is therefore consistent with respiratory metabolism, even in those cells that ferment a significant fraction of this sugar.

To enter the PPP, xylulose needs to be phosphorylated by xylulokinase. Previous studies have shown that increasing XKS1 expression can increase growth rate on xylulose [20]. We therefore examined the expression of XKS1 in our strains. Transition of cells from glucose to xylulose induced XKS1 expression in some strains, while in other strains resulted in repression (**Table S3**). No correlation was observed between the level of XKS1 induction and growth rate or ethanol production, although a moderate negative correlation was observed between absolute expression on xylulose and ethanol production (**Fig. 2D**).

Genes in the PPP pathway displayed a more interesting behavior. Expression of most genes in the oxidative phase of the pathway (upstream of xylulose-phosphate and ribose-phosphate) was inversely correlated with ethanol production, whereas expression of most genes in the non-oxidative phase (utilizing xylulose-phosphate) was positively correlated with ethanol production. Furthermore, expression of genes converting ribose-5-phosphate to PRPP was negatively correlated with ethanol production, while expression of genes producing ribose-5-phosphate (RBK1) was positively correlated with ethanol production. Together, these results suggest that ribose-phosphate may be limiting for xylulose fermentation and that strains which better direct the co-utilization of xylulose-phosphate and ribose-phosphate towards the non-oxidative part of the PPP achieve better xylulose fermentation (**Fig. 2D**).

### Lack of coordination between growth rate and ribosomal-related gene expression

One of the distinguished properties of the yeast transcriptional network is the tight coordination of growth rate with the expression of genes coding for RP and Ribi genes. Similarly, genes involved in the ESR show a correlation with cell growth rate. This correlation is observed when comparing cells growing in a wide variety of naturally occurring environments.

**Figure 3:**
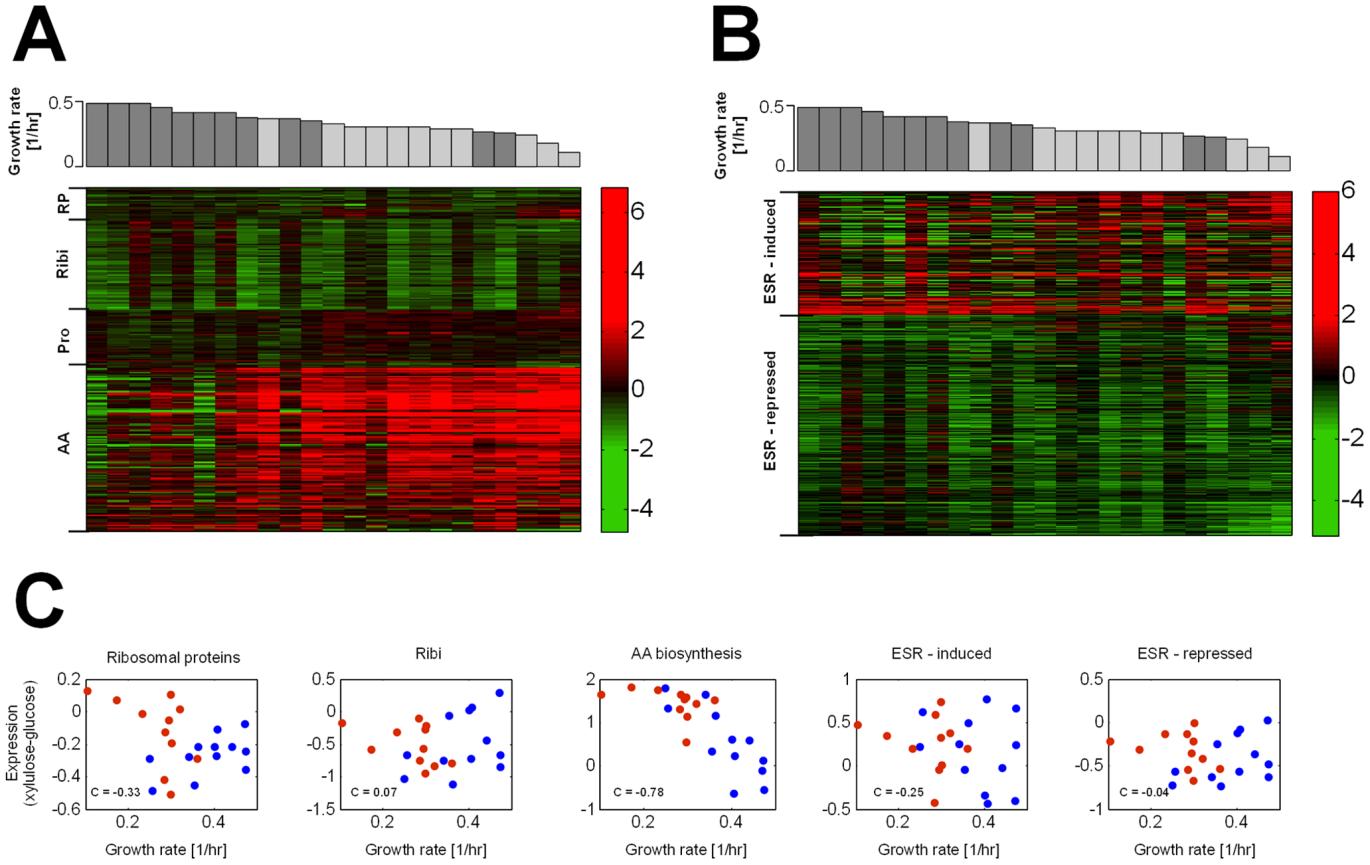
Relation between growth rate and expression of typically growth rate-related genes during growth on xylulose across natural strains. (A,B) Difference (log ratio) between expression on xylulose and on glucose of genes belonging to the RP, Ribi, proteasome (pro) and amino acid biosynthesis (AA) gene groups (A), and genes induced or repressed during the ESR (B). Genes for which data from less than 14 strains existed were omitted. The complete list of genes can be found in Table S2. Each column represents data from an individual strain. Growth rate on xylulose of the respective strain is shown above, for each *S. cerevisiae* (dark gray) and *S. paradoxus* (light gray) strain. The columns are sorted according to growth rate. Same format matrices but for absolute expression levels on glucose and xylulose are shown in Fig. S4A, B and Fig. S5A, B, respectively. (C) Mean difference (log ratio) between expression on xylulose and on glucose over all genes belonging to the RP, Ribi and amino acid biosynthesis (AA) gene groups, as well as genes which are induced and genes which are repressed during the ESR, for each of the 12 *S. cerevisiae* (blue) and 12 *S. paradoxus* (red) strains, vs. growth rate. Same plots but for absolute expression levels on glucose and xylulose are shown in Fig. S4C and Fig. S5C, respectively.

We asked if the same pattern of correlation is observed also across the different strains. As can be seen in **Fig. 3**, a shift from glucose to xylulose resulted in a coordinated change in the expression of all three gene groups. As expected, RP and Ribi were down-regulated in most strains, while the ESR-induced genes were largely induced by the shift. Surprisingly, however, the level of RP and Ribi repression did not correlate with change in growth rate. Similarly, the extent of ESR induction did not correlate with the change in growth rate.

We further examined the absolute expression levels of those three gene groups on glucose and on xylulose, and compared them to growth rates in the respective media (**Fig. S4, S5**). Here too, no correlation between the expression of those gene groups and growth rate was observed.

### Amino-acid biosynthesis gene expression correlates with growth rate on xylulose and in mutants or drug-inhibited cells

We systematically examined for gene groups whose expression correlated with growth rate, using predefined gene groups based on GO categories and transcriptional modules [21]. The single gene group identified showing strong anti-correlation between growth rate and expression was the set of GCN4-regulated genes, required for amino-acid biosynthesis (c = -0.79, **Fig. 3A,C**). This correlation was unique to xylulose and was not observed during growth on glucose.

Complementing their anti-correlation with growth rate, the GCN4-regulated genes were consistently induced upon transfer to xylulose (**Fig. 3A**). Gcn4p is normally induced at the translational level under conditions of amino-acid limitations [22]. Considering the fact that growth on xylulose was performed in media rich in amino-acids (YP-xylulose), the induction of this module was surprising. Furthermore, this gene group was not previously associated with any growth-rate response, as it was not induced during slow growth on carbon sources such as ethanol, glycerol, galactose or fructose. In addition, environmental perturbations which inhibited cell growth did not induce GCN4-dependent genes.

**Figure 4:**
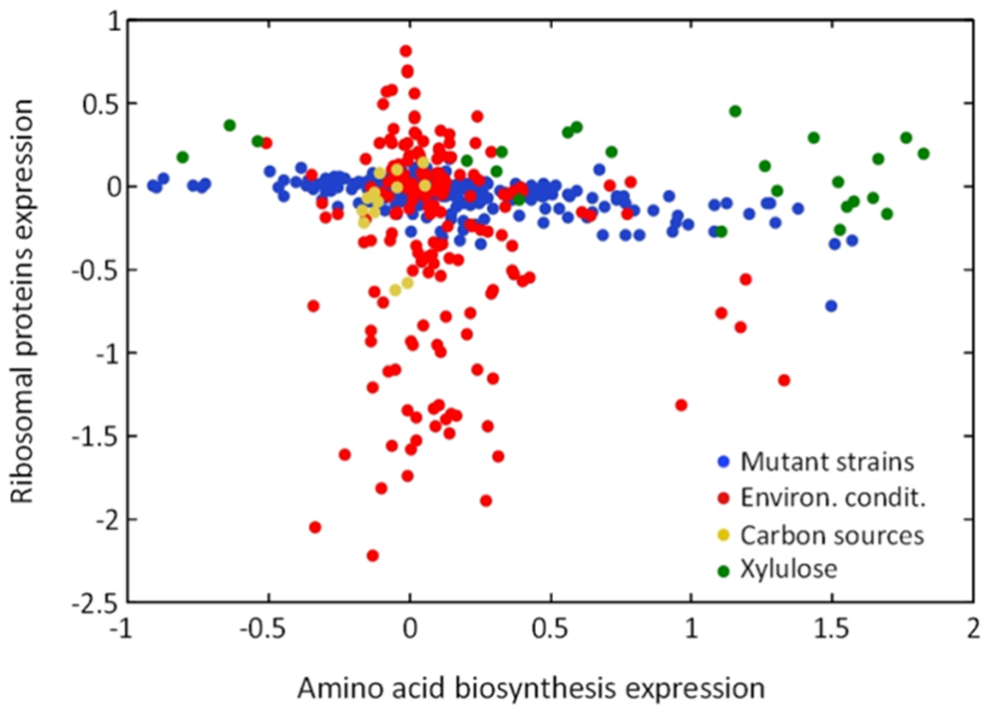
Expression response to various environmental conditions and genetic perturbations. Expression of RP genes vs. amino-acid biogenesis genes for a set of deletion mutation strains and strains treated with various drugs (blue), a laboratory strain grown in various environmental conditions consisting of temperature and osmotic shocks, amino-acid starvation, nitrogen depletion, addition of hydrogen-peroxide/menadione/DTT (red), a wild type strain grown on a variety of carbon sources, including ethanol, galactose, glucose, mannose, raffinose and sucrose (orange) and all 24 strains from our yeast collection grown on xylulose (log ratio between expression on xylulose and on glucose, green). We note that expression data was obtained using different microarrays but are nevertheless compared due to normalization of the data and the fact that expression differences between conditions are used.

We therefore examined the available datasets [21] for additional conditions that similarly modulate the GCN4-dependent program in a growth-related manner. Interestingly, when comparing the expression of the characteristically growth-related genes belonging to the RP and Ribi gene groups with the expression of GCN4-regulated genes, we observed a distinct bi-modal behavior (**Fig. 4**), whereby some conditions that strongly modulated the RP (or Ribi) genes maintained GCN4-dependent gene expression unchanged, while conditions which strongly modulated GCN4-dependent expression did not alter RP (or Ribi) gene expression.

Characterizing the two sets of conditions, we noted that RP genes modulation is predominantly associated with environmental perturbations, while the GCN4-related response is induced in a variety of gene deletion mutants and in strains subjected to drugs [23]. Notably, the deleted genes inducing the GCN4-dependent program were involved in diverse functions, most of which had no apparent connection to amino-acid biosynthesis. The rare cases which combined the two classes of responses, modulating the expression of both the RP/Ribi genes and amino-acid biosynthesis genes, consisted of environmental perturbations leading to amino-acid starvation or nitrogen depletion [7].

Growth rates of most of the deletion mutants were reported, enabling us to examine the correlation of gene expression with cell growth rate. Induction of the GCN4-dependent group was inversely correlated with growth rate (c = −0.36), similar to the aforementioned anti-correlation observed in cells growing on xylulose. Growth rate was also correlated with RP gene expression in this dataset (although those genes were only moderately modulated) but showed no correlation with Ribi gene expression.

## Discussion

Genome-wide expression profiling serves as a central tool for characterizing cellular mechanisms. Thousands of expression profiles are now available describing the cellular transcriptional program in different environments and genetic backgrounds. A key challenge in analyzing such data is distinguishing between specific responses, tuned to the particular perturbation analyzed, and general expression changes resulting from modulation of global cellular phenotype such as cell growth rate.

In the budding yeast, cellular growth rate in different environments is tightly correlated with the expression of ribosome-related genes and with the expression of stress-induced genes. We began this study by asking whether this relation between gene expression and growth rate is also observed when comparing related strains and species. Our results indicate that this is not the case: on neither of the two carbon sources tested – glucose and xylulose – did we observe the expected correlation between growth rate and the expression of ribosome-related genes or the expression of stress-induced genes.

Specifically on xylulose, we observe a strong correlation between growth rate and the expression of the group of amino-acid biosynthesis genes. Examining available gene expression databases, we observed a similar correlation also in a set of deletion mutants. Amino-acid biosynthesis genes are co-regulated by the transcription factor GCN4 whose translation is induced upon starvation to any one of the amino acids. However, in contrast to other metabolic- or translation-related genes, GCN4 is not generally activated in response to environmental stresses. In addition, expression of this gene group does not correlate with cell growth rate under most environments previously tested, with the exception of environmental perturbations specifically related to changes in amino-acid availability.

In light of these results, at least two alternative explanations can be envisioned. First, the lack of typical correlation between ribosome-related gene expression and growth rate when compared between strains growing on xylulose might reflect a general picture of which related strains or species do not display the same pattern of correlation as observed across different environments. According to this possibility, the mechanisms coordinating gene expression and cellular growth upon changing environments are different from those acting on evolved populations. This result is consistent with a recent report which, focusing on hemoglobin as a model system, has shown that physiological and evolutionary adaptation act by modulating distinct sets of parameters [24].

Alternatively, the similar behavior between expression of amino acid biosynthesis genes during growth on xylulose and in a library of gene deletion mutants might suggest the existence of a common underlying regulatory force acting specifically in these conditions. What may be common to wild-type strains growing on xylulose and mutants strains growing on rich media? One possibility is that both sets of perturbations present situations for which cells are not likely to be evolutionary optimized. Xylulose is not available in the wild, and similarly, mutations or drugs are not frequently encountered. Therefore, particularly under these conditions, the typical coordination between ribosome biogenesis gene expression and cell growth might be lacking, leading to an imbalance between the production and use of amino acids, which becomes rate-limiting for growth. Indeed, the well-defined activation of GCN4 is known to be dependent on the accumulation of uncharged tRNA molecules signaling amino-acid depletion [22]. Additional experiments are necessary in order to determine between these two alternatives.

## Materials and Methods

### Strain collection

*S. cerevisiae* strains YPS1009, T7, T73, PW5, Y12, CLIB324, CLIB215, CBS7960 and YJM269 were a kind gift from Dr. Justin Fay of the Genome Institute, Washington Univeristy, St. Louis, USA. *S. cerevisiae* strain EC1118 was purchased from Micha Lerer, Nes Ziona, Israel. *S. cerevisiae* strains S288c and SK1 were present in our lab. All *S.paradoxus* strains were purchased from the National Collection of Yeast Cultures, UK.

### Xylulose production

A mixture of xylulose and xylose was produced in the lab, following the method described in [17]. Briefly, 350 gr of xylose (Sigma-Aldrich) was dissolved in 500 ml water and 20 gr of immobilized Xylose Isomerase (Sigma-Aldrich) was added. This mixture was incubated in 60°C for 24 hours with agitation (300 rpm). The enzyme was inactivated by heating to 100°C and then filtered off. The resulting xylose:xylulose ratio was determined by HPLC analysis.

### Sugar and ethanol analysis

The concentrations of xylulose, xylose and ethanol was measured using an Agilent 1200 series high-performance liquid chromatography system equipped with an anion exchange Bio-Rad HPX-87H column (Bio-Rad, Hercules, CA). The column was eluted with 5 mM sulfuric acid at a flow rate of 0.6 mL/min at 45°C. Media samples were taken for ethanol and sugar analysis at early stationary phase.

### Growth rate measurements

For growth rate measurements on glucose, yeast were inoculated in YPD medium containing 2% glucose. For growth rate measurements on xylulose, yeast were inoculated in YP medium supplemented with the xylose/xylulose mixture described above, to a final concentration of 2% xylulose. Yeast were grown in 96-well plates and incubated in a Tecan Evolution200 robotic incubator with gentle agitation. OD measurements were taken automatically every 30 minutes following a one-minute strong agitation period (1400 rpm) before each measurement. Growth rates were determined by plotting the log(OD) values vs. time. Log phase growth rate and doubling time were calculated from the slope of the linear portion of this curve.

### Array hybridization and data extraction

Strains were grown to mid-log phase in YP-glucose or YP-xylulose medium. Total RNA was extracted using MasterPure Yeast RNA purification Kit (Epicentre).The samples were amplified and labeled using agilent two-color Low Input Quick Amp Labeling Kit, hybridized to custom microarrays and scanned using standard Agilent protocols, reagents, and instruments. The scanned images were analyzed using Agilent's Feature Extraction software. The procedure was performed once for growth on glucose and repeated twice for growth on xylulose. In all experiments, the two colors in a given array were used to label different strains and sometimes strains growing on different sugars as well. Differences between strains were controlled for as described below in normalization method.

### Microarray design and data normalization

Arrays with 60 K probes were used. Each gene had 3–4 different probes on average, and each probe was placed in 3–4 different places in the array, to avoid spatial bias. Probes were chosen to have similar GC content (>98.5% had GC content of 41.67% or 43.44%) and to minimize the distance from the gene's 3′ end and minimize the melting temperature.

Normalization process: The median over the 3–4 repeats was taken for each probe. To normalize the bias of each probe relative to the 3′-end of the gene (likely an effect of reverse transcription) the trend of the intensity differences was plotted as a function of the location distance of the probes of each gene relative to the probe closest to the gene's 3′-end. Normalization was thus performed according to this trend. More specifically, variables dx and dy were defined as follows: for each gene the probe closest to the gene 3′-end (P_last) was first identified, and the distances of all probes relative to P_last were added to define dx. Parallel values in dy consisted of the log ratio of intensities of a probe relative to P_last intensities. The trend of dy as a function of dx was then calculated using the Lowess method, added to the (log) intensities of all probes. Per gene intensity was taken as the median of the different probes. Finally, as all experiments differ slightly in dynamic range, gene values were converted to have the same distribution using percentile normalization.

### Data analysis

When absolute expression levels on either xylulose or glucose are reported, the converted normalized values (described above) were used. For expression differences between xylulose and glucose, converted normalized values on xylulose were divided by the corresponding values on glucose. The ratios between xylulose and glucose of the converted values were also used for calculating correlations with other described data sets. All correlations were calculated using Pearson's linear correlation coefficient (Matlab) and the significance threshold for the correlation was set to α = 0.01.

### Data access

Expression data was deposited into the Gene Expression Omnibus (GEO accession numbers: GSE50124 and GSE50125).

## Supporting Information

Figure S1 **Cell size comparison.**
(PDF)

Figure S2 **Expression of metabolic genes on glucose.**
(PDF)

Figure S3 **Expression of metabolic genes on xylulose.**
(PDF)

Figure S4 **Gene groups expression on glucose.**
(PDF)

Figure S5 **Gene groups expression on xylulose.**
(PDF)

Table S1 **Strains used in this study, their origins, growth rates and ethanol production.**
(XLS)

Table S2 **Gene groups and corresponding genes.**
(XLSX)

Table S3 **Expression ratios log(xylulose/glucose) of all PPP genes, for each of the 24 wt yeast strains, and correlation with ethanol production from xylulose per gene.**
(XLSX)
