# Supplementary material for: Coordination of Gene Expression and Growth-Rate in Natural Populations of Budding Yeast
Source: PLoS One. 2014 Feb 12;9(2):e88801. doi: 10.1371/journal.pone.0088801 (PMC3923061; doi:10.1371/journal.pone.0088801)
Supplement: Figure S1 — Cell size comparison. (PDF) [file pone.0088801.s001.pdf]

Cell size comparison

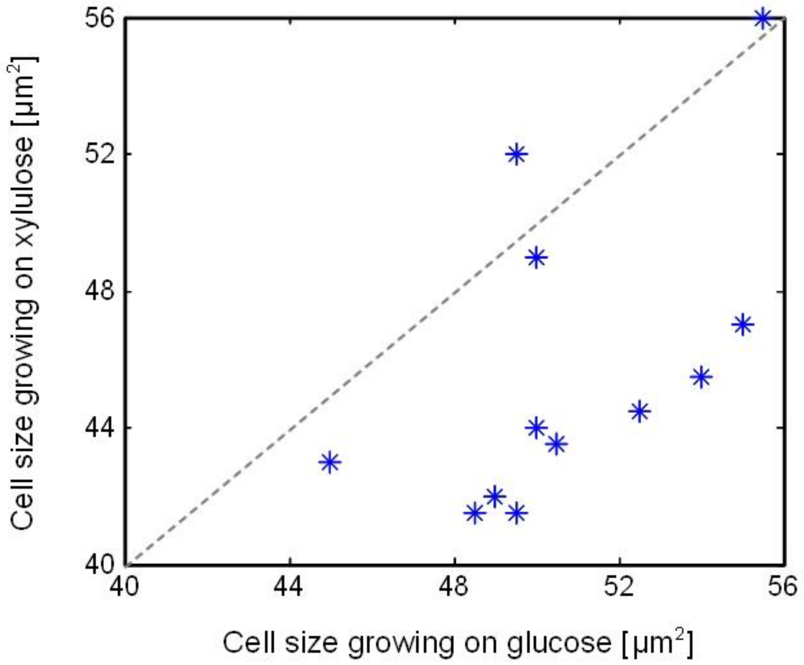

Cell size of all 24 wt yeast strains in our collection during growth on glucose vs. growth on xylulose. Evaluation of cell size was performed using ImageStreamX (Imaging Flow Cytometer, Amnis; www.amnis.com/imagestream.html). Cells were grown in YP medium containing either 2% glucose or xylose/xylulose mixture to a final concentration of 2% xylulose, to mid-log phase, and were directly loaded into the ImageStreamX machine. Data analysis was performed using IDEAS software.
