# Supplementary material for: Coordination of Gene Expression and Growth-Rate in Natural Populations of Budding Yeast
Source: PLoS One. 2014 Feb 12;9(2):e88801. doi: 10.1371/journal.pone.0088801 (PMC3923061; doi:10.1371/journal.pone.0088801)
Supplement: Figure S2 — Expression of metabolic genes on glucose. (PDF) [file pone.0088801.s002.pdf]

Expression of metabolic genes on glucose

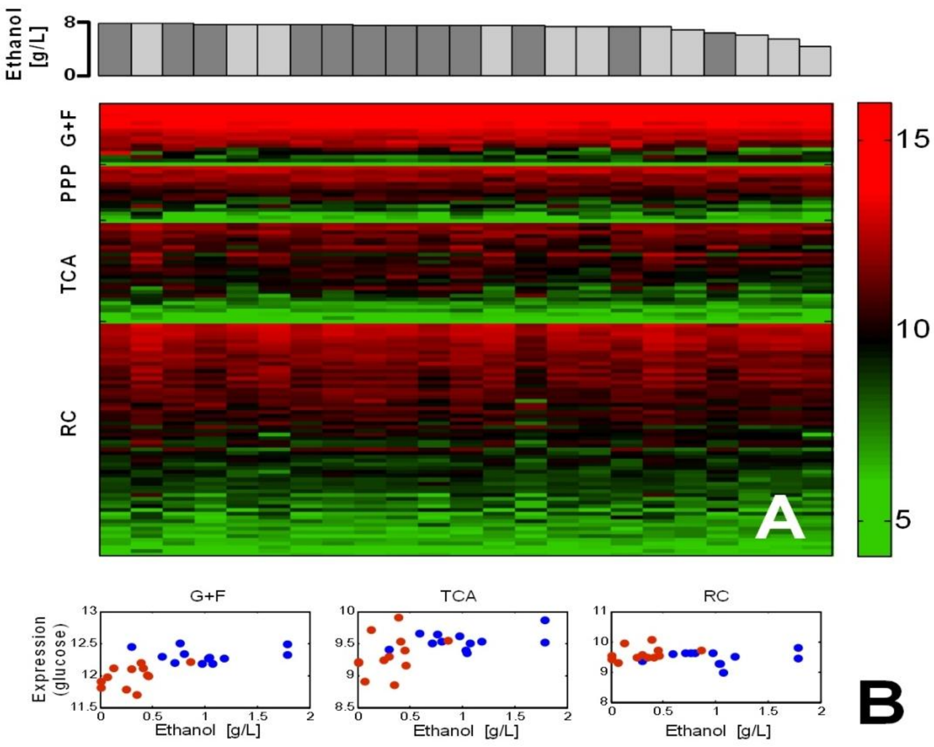

(A) Absolute levels of expression on glucose of genes participating in glycolysis and fermentation (G+F), the PPP, the TCA cycle and the respiratory chain (RC). Genes for which data from less than 14 strains existed were omitted. The complete list of genes can be found in **Additional file 7**. Each column represents data from an individual strain. Ethanol production level on glucose of the respective strain is shown above, for each *S. cerevisiae* (dark gray) and *S. paradoxus* (light gray) strain. The columns are sorted according to the level of ethanol production. (B) Mean absolute levels of expression on glucose over all genes participating in glycolysis and fermentation (G+F), the TCA cycle and the respiratory chain (RC) for each of the 12 *S. cerevisiae* (blue) and 12 *S. paradoxus* (red) strains, vs. ethanol production levels.
