# Supplementary material for: Coordination of Gene Expression and Growth-Rate in Natural Populations of Budding Yeast
Source: PLoS One. 2014 Feb 12;9(2):e88801. doi: 10.1371/journal.pone.0088801 (PMC3923061; doi:10.1371/journal.pone.0088801)
Supplement: Figure S3 — Expression of metabolic genes on xylulose. (PDF) [file pone.0088801.s003.pdf]

Expression of metabolic genes on xylulose

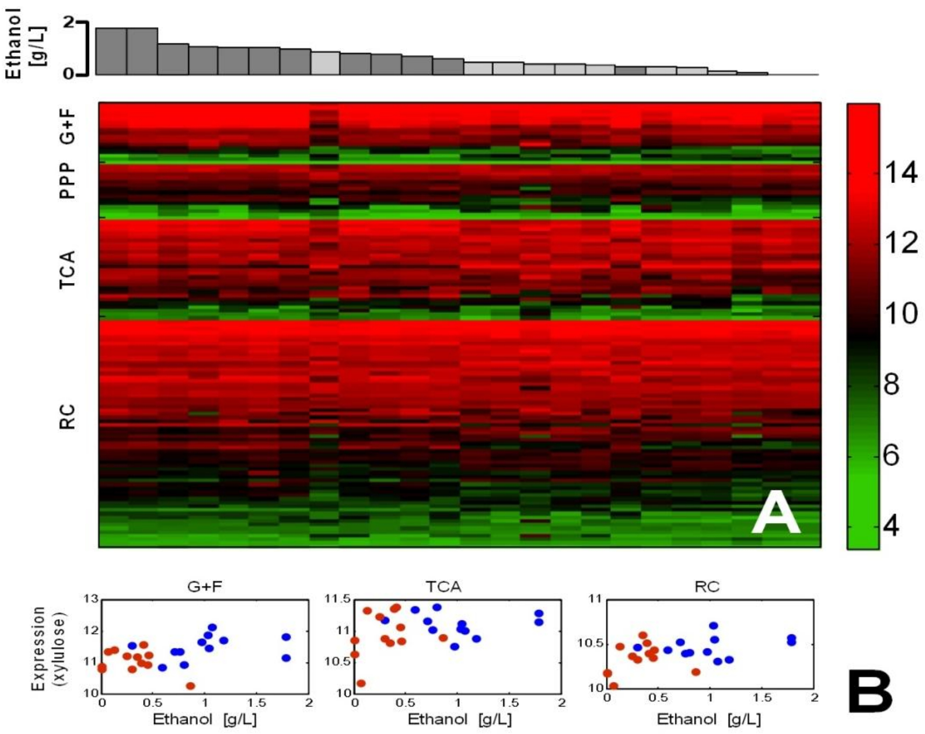

(A) Absolute levels of expression on xylulose of genes participating in glycolysis and fermentation (G+F), the PPP, the TCA cycle and the respiratory chain (RC). Genes for which data from less than 14 strains existed were omitted. The complete list of genes can be found in **Additional file 7**. Each column represents data from an individual strain. Ethanol production level on xylulose of the respective strain is shown above, for each *S. cerevisiae* (dark gray) and *S. paradoxus* (light gray) strain. The columns are sorted according to the level of ethanol production. (B) Mean absolute levels of expression on xylulose over all genes participating in glycolysis and fermentation (G+F), the TCA cycle and the respiratory chain (RC) for each of the 12 *S. cerevisiae* (blue) and 12 *S. paradoxus* (red) strains, vs. ethanol production levels.
