# Supplementary material for: Coordination of Gene Expression and Growth-Rate in Natural Populations of Budding Yeast
Source: PLoS One. 2014 Feb 12;9(2):e88801. doi: 10.1371/journal.pone.0088801 (PMC3923061; doi:10.1371/journal.pone.0088801)
Supplement: Figure S5 — Gene groups expression on xylulose. (PDF) [file pone.0088801.s005.pdf]

Gene groups expression on xylulose

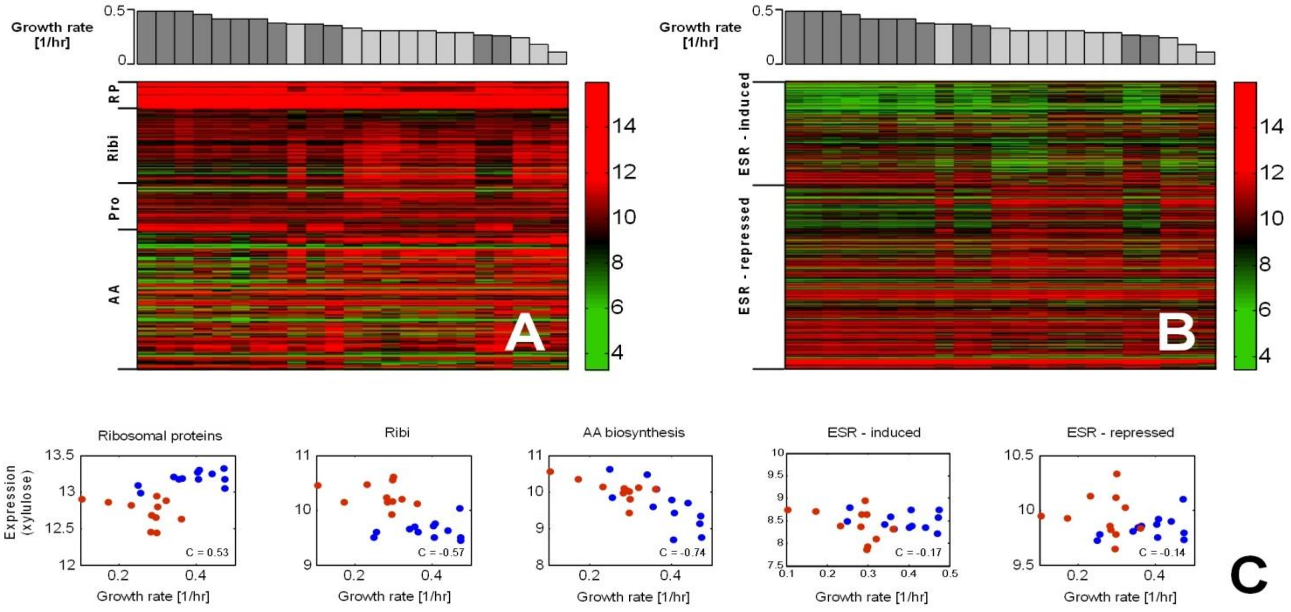

(A,B) Absolute levels of expression on xyluose of genes belonging to the RP, Ribi, proteasome (pro) and amino acid biosynthesis (AA) gene groups (A), and genes induced or repressed during the ESR (B). Genes for which data from less than 14 strains existed were omitted. The complete list of genes can be found in **Additional file 7**. Each column represents data from an individual strain. Growth rate on xylulose of the respective strain is shown above, for each *S. cerevisiae* (dark gray) and *S. paradoxus* (light gray) strain. The columns are sorted according to growth rate. (C) Absolute levels of expression on xylulose over all genes belonging to the RP, Ribi and amino acid biosynthesis (AA) gene groups, as well as genes which are induced and genes which are repressed during the ESR, for each of the 12 *S. cerevisiae* (blue) and 12 *S. paradoxus* (red) strains, vs. growth rate.
